# Using artificial intelligence in medical school admissions screening to decrease inter- and intra-observer variability

**DOI:** 10.1093/jamiaopen/ooad011

**Published:** 2023-02-17

**Authors:** Graham Keir, Willie Hu, Christopher G Filippi, Lisa Ellenbogen, Rona Woldenberg

**Affiliations:** Department of Radiology, Northwell Health, Manhasset, New York, USA; Department of Radiology, Lenox Hill Hospital, Northwell Health, New York, New York, USA; Department of Radiology, Tufts University Medical Center, Boston, Massachusetts, USA; Donald and Barbara Zucker School of Medicine at Hofstra/Northwell, Hempstead, New York, USA; Department of Radiology, Northwell Health, Manhasset, New York, USA; Donald and Barbara Zucker School of Medicine at Hofstra/Northwell, Hempstead, New York, USA

**Keywords:** medical education, admissions, machine learning, artificial intelligence, bias

## Abstract

**Objectives:**

Inter- and intra-observer variability is a concern for medical school admissions. Artificial intelligence (AI) may present an opportunity to apply a fair standard to all applicants systematically and yet maintain sensitivity to nuances that have been a part of traditional screening methods.

**Material and Methods:**

Data from 5 years of medical school applications were retrospectively accrued and analyzed. The applicants (*m* = 22 258 applicants) were split 60%–20%–20% into a training set (*m* = 13 354), validation set (*m* = 4452), and test set (*m* = 4452). An AI model was trained and evaluated with the ground truth being whether a given applicant was invited for an interview. In addition, a “real-world” evaluation was conducted simultaneously within an admissions cycle to observe how it would perform if utilized.

**Results:**

The algorithm had an accuracy of 95% on the training set, 88% on the validation set, and 88% on the test set. The area under the curve of the test set was 0.93. The SHapely Additive exPlanations (SHAP) values demonstrated that the model utilizes features in a concordant manner with current admissions rubrics. By using a combined human and AI evaluation process, the accuracy of the process was demonstrated to be 96% on the “real-world” evaluation with a negative predictive value of 0.97.

**Discussion and Conclusion:**

These results demonstrate the feasibility of an AI approach applied to medical school admissions screening decision-making. Model explainability and supplemental analyses help ensure that the model makes decisions as intended.

## BACKGROUND AND SIGNIFICANCE

Determining which applicants will have the privilege of becoming future physicians is an important and difficult task. The choices have long-lasting implications for not only the applicants but all of society. Unfortunately, medical school admissions is also a lengthy, laborious, and time-consuming process. Many measures are undertaken to standardize the evaluation process, but the fact remains that there is significant inter- and intra-observer variability amongst applicant screeners. Humans are inherently inconsistent evaluators who can be subject to external influences. In one study, judges sentenced juvenile defendants to harsher sentences when their local football team experienced an unexpected loss in the preceding week.^1^ Machine learning models are not subject to the vicissitudes of life and will consistently provide the same output for a given input.

The number of medical school applicants has also been growing at a break-neck pace, increasing 86% over the last 2 decades.^2^ Specifically, our institution, the Zucker School of Medicine at Hofstra/Northwell (ZSOM) has been receiving in excess of 5000 applications per year for a final class size of approximately 100 students. The medical school admissions workflow starts with screening each of the 5000 applicants to determine which of the approximately 700–800 applicants to interview. Of these, approximately 300 will be given an admission offer, and 99 will matriculate. The screening process is intended to be a holistic measure of each applicant, utilizing elements including but not limited to: grade point averages (GPA), Medical College Admissions Test (MCAT), undergraduate institution, research experience, extracurricular activities, socioeconomic factors, personal statement, and letters of recommendation. A committee of ZSOM faculty members then reviews each applicant to decide those to invite for interview, based on the above criteria; and only following a relatively comprehensive interview day and voting process are acceptances issued.

The process of screening a large number of applications for interview is very time-intensive. If we make a simplified assumption of each application taking one faculty member approximately 20 min to screen, for 5000 applicants this equates to 1665 person-hours of work. As this is a data-rich process of sorting applicants into 1 of 2 categories, i.e. offer interview or deny interview, it is a process that may be ideally suited for the application of a machine learning algorithm.

Utilizing machine learning in personnel selection is already somewhat widespread, although its frequency of use may vary with the specific industry in question. Machine learning has been at least anecdotally reported to be used in screening job applicants’ resumes to the point where articles have begun giving advice to applicants on how to get past artificial intelligence (AI) screeners.^3^^,^^4^ To our knowledge, machine learning is not currently widely used within the medical education selection process. However, Burk-Rafel et al^5^ have recently developed and proposed a machine learning-based tool for use in the medical residency application process.

One particular concern of inter- and intra-observer variability is the role that bias may play in the process. When using the term “bias,” we mean prejudice or unfair inclination for or against something, not in the statistical meaning of the word “bias.” Bias in the admissions process can come from many sources. Some examples include racial/demographic information, gender, ethnic names, applicant photos, regional bias, or institutional bias. One study of applications to radiology residency demonstrated discrimination against facially unattractive and obese applicants.^6^ AI has been known to incorporate and even amplify bias already present in data. When the popular publicly-available word2vec embedding^7^ was trained on a large corpus of online news texts, it outputted that “man is to computer programmer as woman is to: *homemaker.*” Even more relevant to our discussion, when asked to solve the analogy “Father is to doctor as mother is to:,” the model outputted “*nurse*.” Bolukbasi et al^8^ therefore developed a methodology for modifying word embeddings to remove gender stereotypes. Racial bias has been detected in an algorithm to forecast criminal behavior, a model to support healthcare decisions, and within search engines and recommendation/recommender systems.^9^

A hypothetically fair algorithm can become biased over time. An algorithm that works fairly in one context could learn biases as it is used in another context, incorporating feedback loops based on learning from its own predictions.^10^ Automation bias can also occur in which a tool intended as decision support becomes the de facto decision maker as humans defer to the predictions over time.^11^

A simple way to prevent a machine learning system from internalizing bias is to not provide it with the information that would allow it to learn biased representations. By not providing an applicant’s name, the model cannot learn to associate a negative connotation with ethnically-identifying names. Not providing the place of birth or current zip code prevents the model from learning to prejudice against people from a certain city, state, or socioeconomic background. When we only provide information that is relevant to decision-making, we can remove opportunities for the algorithm to become biased in the learning process.

Biased representations can still be learned in unpredictable ways, so carefully curating the information fed into the algorithm is not sufficient. Another way to detect and route out bias is the concept of model explainability. The black-box nature of most machine learning algorithms is one of the primary opportunities for the inadvertent learning of unplanned associations, and this prevents acceptance by end-users or those to whom the algorithm is applied. By examining how a model makes decisions and what factors are most predictive, we can look for unintended consequences and correct them. Although we could print out every single decision tree that was used to generate our model, it would be much too complicated to understand on an intuitive level. Instead, we can generate a much simpler explanation model which is an approximation of our original model. SHapley Additive exPlanation (SHAP) values were introduced by Lundberg and Lee^12^ in 2017 as an approach to explaining the output of a machine learning model. They draw on the concepts introduced by Lloyd Shapley in the field of cooperative game theory as a means of calculating how much each individual in a coalition of players is responsible for a total surplus.

There are cases where we intentionally incorporate “bias” into our algorithm. For example, it has been an important goal of medical school admissions committees to promote diversity within a medical school class. One benefit is that it exposes students to different information, value systems, and perspectives to promote cultural sensitivity. A survey of Harvard and UCSF medical students found that contact with diverse peers greatly enhanced their educational experience.^13^ Second, it promotes the expansion of health care to traditionally underserved communities. Underrepresented minority physicians are more likely to serve their communities than their majority counterparts.^14–16^ Also, minorities in North America frequently choose physicians of their own race and rate their physicians’ care as more participatory.^17^ Therefore, bias is intentionally incorporated for not only the benefit of the student body but also our collective health.

The goal of this project is (1) to develop and evaluate an AI algorithmic approach to the evaluation of medical school applications and to (2) analyze the effect on disadvantaged groups. We hypothesized that an AI algorithmic approach will be feasible and that explanatory models and subgroup analysis can help avoid pitfalls such as the marginalization of specific populations.

## MATERIALS

Exempt status was granted by our Institutional Review Board to undertake this study. Five consecutive years of data from medical school applications were retrospectively accrued and analyzed. There were 2 types of data available. One type of data was qualitative, which included volunteerism, professional experience, leadership, scholarly activities, and research publications. The other type of data used was quantitative. This included MCAT scores, GPA, underrepresented minority status, the schools attended, and United States citizenship. From these 2 sources of data, there were 20 features/inputs that were chosen for the model (see Table 1). The choices of which features/inputs to include were based on rubrics that were provided by the admissions committee that were currently used in decision-making, as well as by testing out different features to observe the effect on the model performance (using the Training and Validation sets). Recommendation letters and personal statements were not available due to the technical difficulty of obtaining them from the application system and the challenges of keeping the information anonymous.

**Table 1.** List of features that were used to develop the algorithm

Features
Application year
Date of submission
Total number of hours spent on volunteer activities
Total number of hours spent doing research
Total number of peer-reviewed publications
Total number of scientific presentations
Total number of hours in leadership activities
Total number of hours spent shadowing in a healthcare setting
Highest MCAT score
Science grade point average (GPA)
Total grade point average (GPA)
United States citizen
Socioeconomic indicator
History of Military Service
First generation to attend college
African American
Latino
Native American
Connection to the institution
History of an institutional action against the applicant

The initial data consisted of 27 841 applicants. 5555 applicants were removed because there was no specific data on the interview decision. This was often because the candidate withdrew their application before the admissions committee had an opportunity to evaluate them. An additional 28 applicants were removed because they had incomplete data, such as no MCAT score listed.

**Figure 1.**
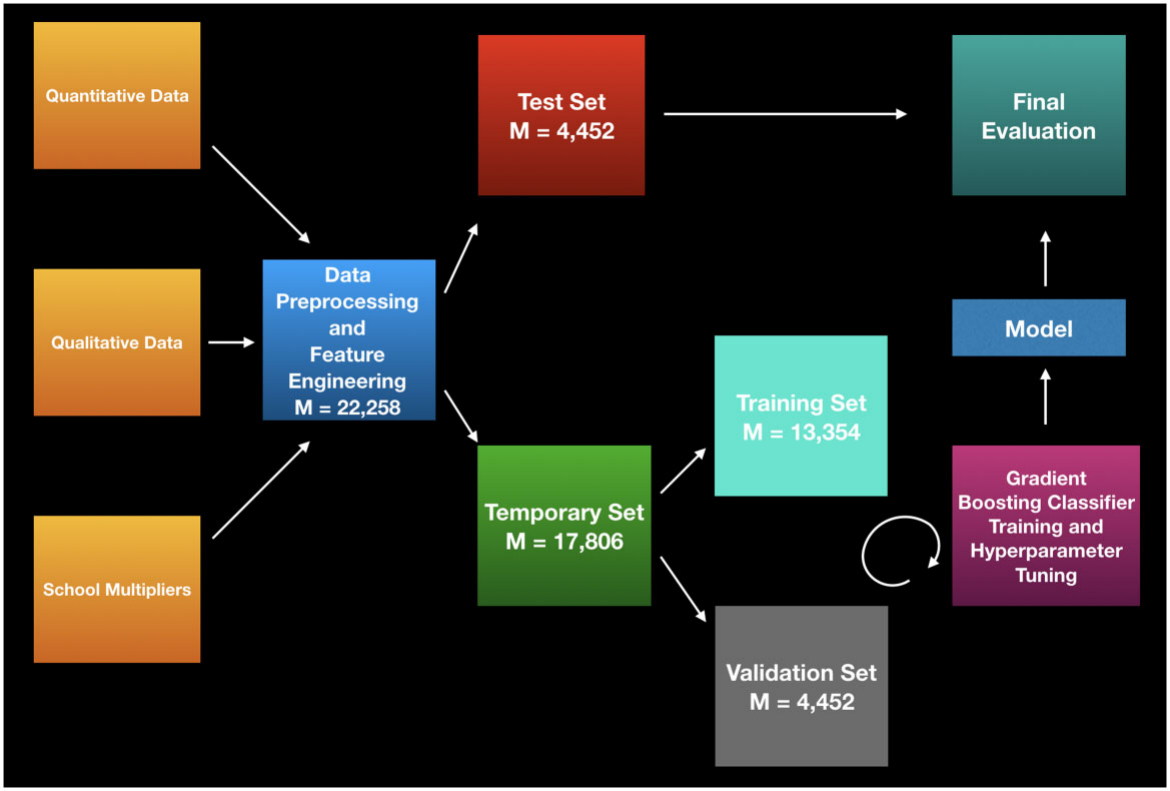
Data pipeline for the training, validation, and final evaluation of the algorithm.

The remaining applicants (*m* = 22 258 applicants) were split 60%–20%–20% into a training set (*m* = 13 354), validation set (*m* = 4452), and test set (*m* = 4452). A model was built consisting of data preprocessing, feature generation, and predictive modeling. An AI algorithm was trained on the training set, parameters were tuned according to the validation set, and performance was evaluated on a “hold-out” test set which was not used to train or validate the model. Ground truth was set as to whether or not a given applicant had been invited for an interview. The code was written in Python 3.7 (https://www.python.org/downloads/release/python-370/) and predictive modeling was performed using XGBoost^18^ (https://xgboost.readthedocs.io/en/latest/), an ensemble tree-based learner with gradient boosting. Hyperparameter tuning was optimized on the validation set using binary cross-entropy and F1 scores. Figure 1 depicts a graphical representation of the data pipeline. After the model was trained and optimized, the SHAP values were calculated to determine the impact of individual features on the model’s final prediction.

Following this, a “real-world” trial was simulated in which the algorithm was run on a single year’s application pool (*m* = 6092) for the admission cycle of 2020–2021. Before this trial, thresholds were chosen to put the applicants into 1 of 3 groups: “reject,” “interview,” or “defer judgment.” These thresholds were chosen by searching for the thresholds that would give the best trade-off between accuracy and F1 score based on the data obtained in the initial pipeline validation and test sets. In the “defer judgment” group, the application was given to a human evaluator as the algorithm had indicated that it lacked confidence in the accuracy of its prediction. Supplemental analyses were conducted on this data to ensure the model was free of significant racial biases. Model performance was evaluated separately for inviting and rejecting applicants as the 2 modalities produced significantly different results.

## RESULTS

Analysis of the Training, Validation, and Test sets demonstrated that there were no significant differences between the populations, specifically in the percentage of gender and minority representation. The algorithm had an accuracy of 95% on the training set, 88% on the validation set, and 88% on the test set. The precision score and recall respectively were 0.80 and 0.92 on the training set, 0.63 and 0.76 on the validation set, and 0.63 and 0.74 on the test set. The F1 score was 0.86 on the training set, 0.69 on the validation set, and 0.68 on the test set. The area under the curve of the test set was 0.93.

A second test set using a cohort from 2020 to 2021 demonstrated the following demographic data as seen in Table 2. When combined with human evaluators into a “real-world” trial, a total of 1689 samples were put in the “defer judgment” group. When the results of the algorithm and human evaluations were combined, the entire process had an accuracy of 96%. The precision score and recall respectively were 0.90 and 0.85. The F1 score and negative predictive value were respectively 0.875 and 0.97. A total of 412 invites were given by the algorithm and 583 invites were given by a human.

**Table 2.** Demographics of the applicants invited and rejected in one test set based on the cohort from the 2020–2021 application cycle

	Invited	Rejected
Total	*N* = 1582	*N* = 4509
Female	784 (49.6%)	2493 (55.3%)
Native American	3 (0.2%)	33 (0.7%)
African American	146 (9.2%)	350 (7.8%)
Latino	208 (13.1%)	333 (7.4%)

## DISCUSSION

**Figure 2.**
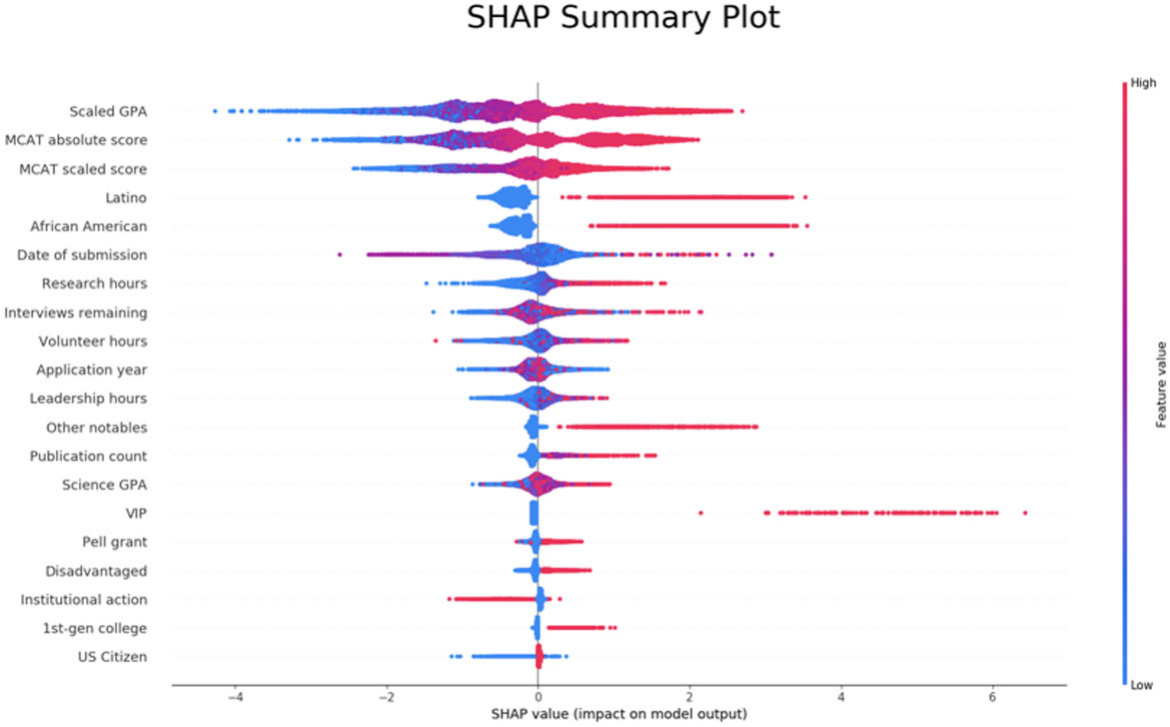
SHAP summary plot for the features that were found to be most important to the model. As the feature value increases, it moves from blue to red color, and the impact on the model is demonstrated as the SHAP value, measured along the *X* axis. “Adj BCPM GPA” was the grade point average specifically for science classes (Biology, Chemistry, Physics, Math), adjusted using the relative rating of the institution where the student studied. A “VIP” was an applicant who had a strong connection to the institution, such as a family member who worked for the medical school and/or healthcare system. “Date of Submission” was calculated as the number of days from the opening of the application cycle to the date when their application was finalized and reviewed.

These results demonstrate that AI can be applied to medical school admissions screening decisions with good predictive ability. A combination of human and AI evaluation can improve the performance while still greatly reducing the number of human evaluations and allowing for focused attention on specific subsets. By applying the same model to every applicant, we have sought to reduce variability in the process. Though reducing variability may help to eliminate individual bias, it does not eliminate systemic bias—it might in fact have the opposite effect. We must remain extremely vigilant to detect and prevent systemic bias. By providing the algorithm with only predefined information necessary for decision-making, we minimize the opportunity for the algorithm to learn such biases. The SHAP model explainability techniques can help ensure that the model is functioning as intended. An evaluation of the SHAP summary plot demonstrates that for each feature, the impact on the model is as to be expected (Figure 2). For instance, as the value for MCAT score increases (becomes more red), we see that it has an increasingly positive effect on the model output. For the Latino category, a high value (values given as 0 for non-Latino or 1 for Latino), a high value has a positive effect, whereas a low value has a neutral effect.

**Figure 3.**
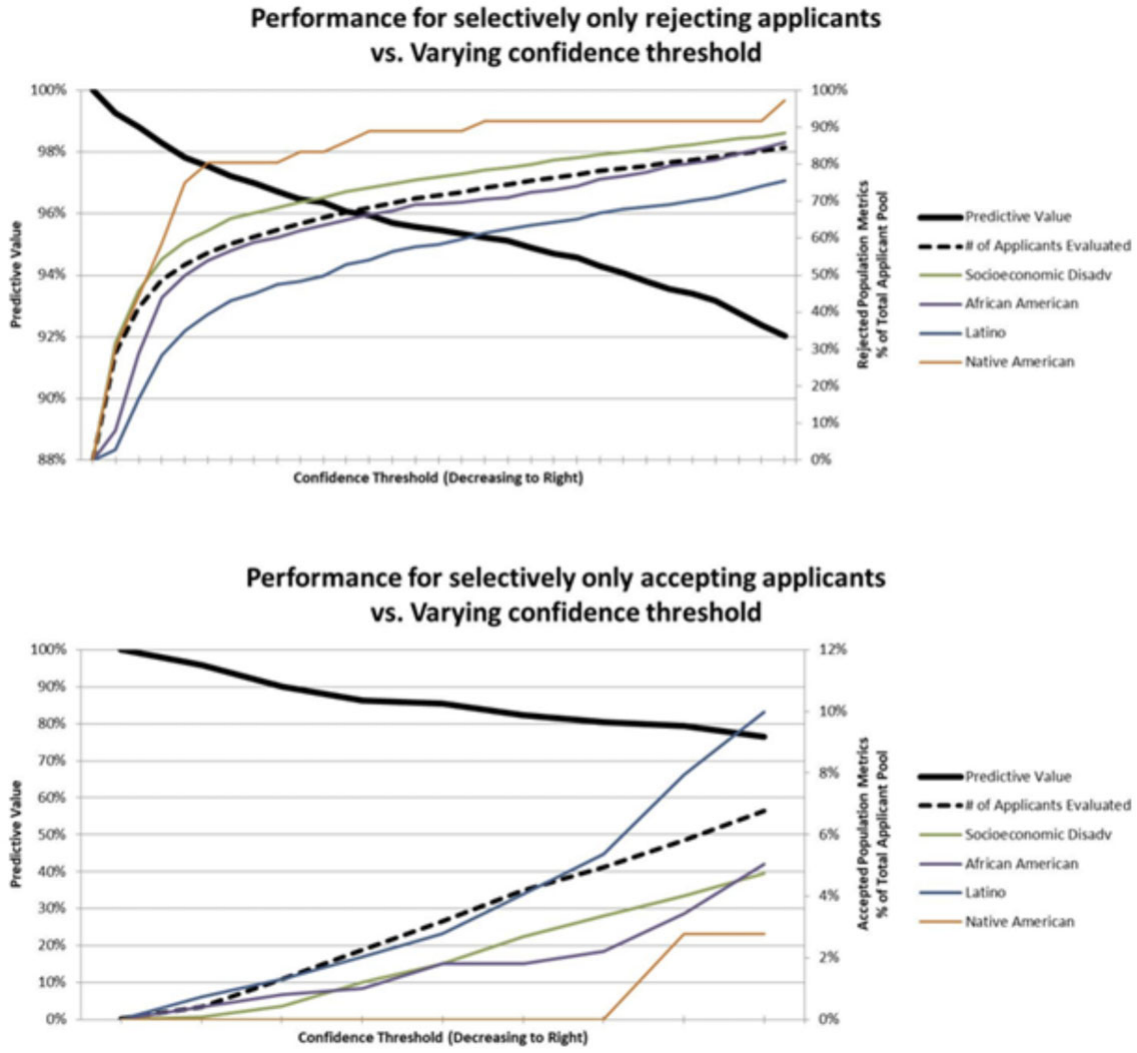
(Top and Bottom) Performance was evaluated separately for inviting and rejecting applicants as the 2 modalities produced significantly different results. The first chart (Top) demonstrates that as the required confidence level to reject applicants decreases, predictive value drops but the total number of rejected applicants increases. The second chart (Bottom) demonstrates that the model performs less well overall for inviting applicants. In terms of performance with underprivileged minorities, Latinos are invited in proportionately equal or greater numbers, while African Americans are somewhat less likely to be invited.

Specific subgroup analyses were performed to investigate how the model performs for underrepresented minorities when recommending which applicants to reject (Figure 3A), and when recommending which applicants to interview (Figure 3B). In the setting of applicant rejection, Latinos and African Americans are both rejected in proportionately fewer numbers when compared to the total applicant pool. In the setting of applicant invitations for interviews, the model invites Latinos in proportionately equal or greater numbers than overall applicants, while African Americans are somewhat less likely to be invited. An important caveat is that these are simply overall numbers of subgroups and do not in any way take into account the qualities of the applicants. However, this data is important to examine as we do not want the algorithm to disadvantage any specific group. The model appears to be better suited as a screening test to recommend which applicants to reject rather than to recommend which applicants to invite. This agrees with what is observed with the overall performance, in which the negative predictive value is substantially higher than the positive predictive value. In addition, Native Americans appear to be rejected in a higher proportion and invited in a lower proportion compared with the overall applicant pool. The simplest explanation for this is likely that the number of data points for Native Americans is extremely low (36 out of 6092 applicants) and machine learning techniques can only be valid when applied to large datasets. Regardless of the source of the error, the lower performance suggests that in a real-world setting, a human evaluator should review all Native American applications. These applications are few in number and would not be excessively burdensome to the process, even if more than one reviewer were to cross-check each other’s work. Although personal biases may exist in the evaluators, at least this methodology would ensure that this population was not being specifically disadvantaged by the algorithm.

There were several limitations to this study. First, the final interview decision was used as the ground truth. In medical school admissions, there is no gold standard. There is no specific combination of qualities that makes an ideal doctor. Institutions vary in what they look for in their applicants, and this is often mission specific. In fact, Harvard Medical School recently announced that they would withdraw from US News and World Report Rankings concluding that the unintended consequences of the ranking system encouraged them to select applicants and financial aid awards in order to boost rankings rather than “nobler objectives.”^19^ Further, qualities that make one doctor an excellent pathologist may also make them a terrible surgeon or vice versa. There have been some suggestions that admissions should focus on the downstream outcomes, such as Alpha Omega Alpha Society (AOA) or Gold Humanism Society attainment, number of medical school honors, board scores, or eventual residency match outcomes. Given the much smaller numbers of medical students compared with applicants, it would be difficult to obtain sufficient data to train any sort of meaningful machine learning model using this data. Further, this approach might result in a more homogenous group of applicants selected. Underlying these choices, there is an assumption that someone achieving more honors or applying for a more competitive specialty is somehow a better doctor, whereas experience tells us that this may be far from the truth. Further, there are potential societal consequences if we begin selecting only for traits that lead someone into a medical or surgical specialty versus the traits that lead someone into primary care.

**Figure 4.**
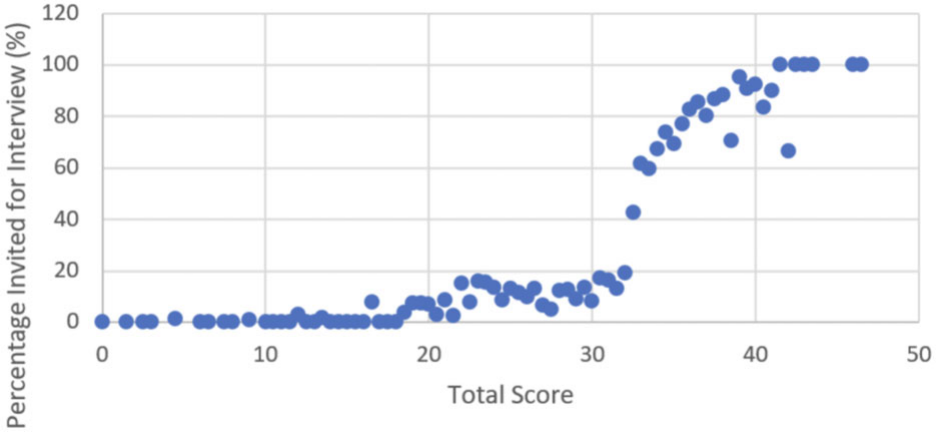
The percentage of applicants invited for an interview was plotted for each score totaled by evaluators for a single application season (2019–2020). There is a gradation as the scores increase, rather than a specific cutoff above which every applicant is invited.

Interview decisions are inherently variable and may in contain the biases we are trying to eliminate. One evaluator may decide to invite the same applicant that another evaluator would reject, given the same score. This variability is made clear in Figure 4, where the percentages of applicants invited with a given score are plotted for a single application season. Toward the evaluations in the 30–40 range, half of the applicants would be invited whereas another half would be rejected. The goal of the algorithm then is not to fit every idiosyncrasy of the decision-making, but rather to pull out general trends. In the hypothetical scenario in which an algorithm determines the perfect trends to maximize predictive ability, we would not expect the accuracy to be 100%. We would not want the model to learn to fit incorrect examples. For this reason, given a dataset of imperfect labels, we cannot determine what the optimal performance should be. However, we can get a sense of how well the algorithm is performing by looking at how the algorithm uses specific values to affect its prediction. An exploration of the SHAP diagram (Figure 2) demonstrates that the model is making decisions for the right reasons.

A second limitation of this study was the data itself. The recommendation letters and personal statements were not available. According to the rubric provided by the medical school admissions committee, these factors play a considerable role in the scoring of an applicant. Of course, these letters will be read (and are often more meaningful) when the applicant comes for an interview. Yet without these letters and essays, there are 2 categories of data to which the model does not have access. These documents may or may not serve as a source of individual bias for evaluators as they are able to glean more biographical information about an applicant through the personal statement. In fact, in a study of urology applicants, significant linguistic differences were discovered in letters of recommendation for men versus women.^20^ The authors suggested that this may have an effect on the recruitment of female residents to the specialty. Similar findings have been found in other medical specialties.^21^^,^^22^ At many institutions, including our own, there has been a recent movement to reduce the emphasis on personal statements and letters of reference, which both have limited value in differentiating applicants or predicting medical school performance.^23^ Our institution has recently begun using CASPer (Computer-Based Assessment for Sampling Personal Characteristics) as a standardized mechanism of assessing personal and professional attributes. CASPer is a 12-section test, with 8 video-based sections and 4 word-based sections. In each section, the applicant is presented with a scenario and then asked to answer 3 questions. Raters assign a score to each section on a 1–9 Likert-type scale, seeing only one section per applicant. These scores are then averaged into an overall score. As each evaluator only sees one section, and is blinded to the identity of the applicant, there is a reduced opportunity for bias in the process.^24^ Similarly, the Association of American Medical Colleges (AAMC) offers the PREview™, a professional readiness exam designed to assess examinees’ pre-professional knowledge in 8 core competencies.^25^

In addition, the recent public introduction of advanced generative AI language models, such as ChatGPT (Generative Pre-trained Transformer) Chatbot released by OpenAI^26^ questions the role that personal statements should play in the admissions process going forward. As Jim Jump, the past president of the National Association for College Admissions Counseling writes for Inside Higher Ed:

*“The low-hanging-fruit answer is that it is clearly unethical for a student to submit an essay written by ChatGPT. The more complicated question is whether it is unethical for a college to require an application essay or make the essay a significant factor in evaluating a student’s application. How can you use an application essay to help make admission decisions when you can’t tell whether the student actually wrote the essay?”*^27^

The same ethical concerns apply to medical school admissions. The medical admissions community is just coming to terms with these new generative AI language models and there is as of yet no consensus as to what these developments herald for the medical student personal statement. A third limitation is the inherent variability of the admissions process. This is well demonstrated in Figure 4. Looking at the medical school statistics from the admissions year 2020, there was no consistent score threshold to which an applicant was invited. Therefore, had our model perfectly calculated an applicant's score, the performance would still not be 100%. Regardless, the model derives its utility not by perfectly fitting the data, but by its ability to generalize and pull out trends. Were the model to perfectly fit the data, we would be incorporating individual biases of the screeners, which we explicitly want to avoid. Rather, we seek to build a model that incorporates the over-arching values of the collective admissions body without individual biases.

The algorithm performance must also be put into the proper perspective when considering the role of AI in the admissions process. This algorithm was developed as a screening algorithm for applicants to interview. Once an applicant is brought for an interview, a human being would still look through the complete application and determine whom to accept, which is the normal, standard operating procedure. Therefore, the algorithm is still subjected to significant human oversight. Medical schools interview significantly more applicants than spots available. Therefore, a few wrong decisions can easily be handled by the admissions committee. In addition, though the algorithm was evaluated using a binary classification of invite versus do not invite, this is not necessarily how such an algorithm would be used. Depending on the needs of the admissions committee, the thresholds could be varied to optimize the trade-off between performance and the number of candidates evaluated in person.

The greatest value of this algorithmic approach is perhaps not in its hypothetical ability to improve efficiency or as a human replacement. By modeling the application decisions using a machine learning context, we are able to learn more about the admissions process itself. Looking at the model explainability outputs as well as the specific subgroup analysis, we are able to see what the model has learned to prioritize in the admissions process. For example, we may say that we prioritize having a diverse student body, but the way model learns to make choices may indicate whether or not we actually carry out these priorities and how consistently.

AI is finding its way into more and more settings. It is the opinion of the authors that it is only a matter of time before AI becomes commonplace in admissions decisions, including those of medical schools. Therefore, we feel it is imperative to thoroughly scrutinize algorithms to ensure that bias is not being preserved unintentionally or even amplified. If done correctly, there is an opportunity to make the admissions process fairer and more standardized. Most important, an AI model will always output the same decision for a given set of inputs, and this will not change based on the time of day, day of the week, or assumptions made by glancing at an applicant’s photograph or personal statement. Applying the same standard to every applicant means that no applicant will be privileged by characteristics that are not directly relevant to the admissions process. This study serves as a proof-of-concept that a machine learning algorithm can be successfully trained and utilized with good predictive ability. Some safeguards may help ensure that the algorithm is making decisions for the right reasons and not disadvantaging certain groups. This approach not only holds promise as a method for reducing inter- and intra-observer variability in the admissions process but also as a means of evaluating the admissions process itself and determining how consistently the criteria are applied. Although we recognize that there are inherent limitations to our approach, we feel that it is important to engage in this type of research and start a conversation on how machine learning algorithms may supplement or improve the process of medical school admissions.

## Data Availability

Code is available through the GitHub repository https://github.com/grahamkeir/JAMIA-Submission. The data underlying this article cannot be shared publicly due to the inherent biographical nature of the data. Limited, non-identifying data will be shared on reasonable request to the corresponding author.

## PREVIOUS PRESENTATIONS

Keir G, Hu W, Filippi C, Ellenbogen L, Fazio C, Miller L, Woldenberg R. (August 27, 2020). Reducing Bias in Medical School Admissions using Artificial Intelligence [Poster presented]. Eastern Neuroradiological Society/Virtual, USA.

Keir G, Hu W, Filippi C, Ellenbogen L, Fazio C, Miller L, Woldenberg R. (May 22, 2021). Using Artificial Intelligence to Reduce Bias in Medical School Admissions [Poster presented]. American Society of Neuroradiology/Virtual, USA.
